# Minimal-Risk Seed Heteromorphism: Proportions of Seed Morphs for Optimal Risk-Averse Heteromorphic Strategies

**DOI:** 10.3389/fpls.2018.01412

**Published:** 2018-10-01

**Authors:** P. William Hughes

**Affiliations:** ^1^Department of Plant Breeding and Genetics, Max Planck Institute for Plant Breeding Research, Cologne, Germany; ^2^Botanical Institute, University of Cologne, Cologne, Germany

**Keywords:** risk aversion, risk spreading, seed dimorphism, seed heteromorphism, seed morphs

## Abstract

Seed heteromorphism is the reproductive strategy characterized by the simultaneous production of multiple seed types. While comparing heteromorphic to monomorphic strategies is mathematically simple, there is no explicit test for assessing which ratio of seed morphs minimizes fitness variance, and hence offers a basis for comparing different heteromorphic strategies. Such a test may be particularly valuable when more than two distinct morphs are present, since many strategies may have equivalent geometric fitnesses. As noted by Gillespie (1974), in these cases avoiding rare but evolutionarily important instances of severe reductions in fitness involves the minimization of variation in fitness—i.e., risk. Here I compute the optimal proportions of two or more seed morphs for heteromorphic strategies that either: (1) minimize total fitness variance; or (2) maximize the fitness-risk ratio—i.e., the “extra” fitness accrued per unit of “extra” fitness variance. This work thereby provides a testable null hypothesis to estimate the optimal frequencies of seed morphs when multiple heteromorphic strategies have evolved in environments with severe fitness risks. Moreover, it also permits the calculation of expected seed morph frequencies when more than two seed morphs are produced.

## Introduction

Seed heteromorphism (also termed seed polymorphism or seed dimorphism) is the evolutionary strategy where a plant is capable of producing two or more distinct types of seed, which may differ in morphology, ripening, dormancy, seed size, dispersal, or germination time (Venable, 1985; Venable and Levin, 1985; Baskin and Baskin, 1998; Clauss and Venable, 2000; Imbert, 2002; Wang et al., 2010). Producing multiple seed types, each with distinctive adaptations, allows plants to specialize in more than one type of environment, and helps offset fitness losses when environmental variation is uncertain. The evolution of heteromorphic seeds has occurred in many clades: recent estimates count as many as 292 species, in 26 families, displaying this behavior (Imbert, 2002; Wang et al., 2010; Auld and Rubio, 2013).

Seed heteromorphism has been shown by a wide array of empirical and theoretical work to be an effective bet-hedging strategy (Harper et al., 1970; Stebbins, 1974; Westoby, 1981; Kaplan and Cooper, 1984; Lloyd, 1984; Wang et al., 2015b). In unpredictable environments, bet-hedging strategies obtain a long-term selective advantage by minimizing temporal variation in fitness, despite that this usually entails a reduction in arithmetic fitness over the short term (Cohen, 1966; Seger and Brockmann, 1987; Philippi and Seger, 1989). Thus bet-hedging strategies are said to maximize geometric mean fitness (e.g., “the nth root of the product of n fitness values,” p. 1601, Simons, 2011) rather than arithmetic mean fitness. Bet-hedging strategies are thought to be able to take one of two main forms: conservative and diversified (Philippi and Seger, 1989). Conservative bet-hedging strategies produce phenotypes that are “safer” than the arithmetically optimal strategy, as a hedge against severe but unpredictable events. For example, the perennial *Carlina vulgaris* flowers earlier in its life than would be strictly optimal, presumably to avoid uncommon but severe “high mortality years” (Rees et al., 2004, 2006). In this case the plant reproduces earlier than would be strictly optimal in many years, just to avoid reproducing too late in years when the mortality costs would be prohibitive. In contrast, diversifying bet-hedging strategies distribute risk among two or more phenotypes, in order to minimize the variance in fitness through time and to ensure that total reproductive failure does not occur (Cohen, 1966; Slatkin, 1974; Seger and Brockmann, 1987; Philippi and Seger, 1989; Beaumont et al., 2009; Simons, 2011; Gremer and Venable, 2014). A diversification strategy for seed heteromorphism may show suboptimal fitness for one of the seed morphs over a short time scale but is expected to maximize geometric fitness over many generations by expressing a stable ratio of seed morphs that prevents fitness loss from rare but unpredictable events.

The high-risk—low-risk (HRLR) pattern is the most common documented pattern of seed heteromorphism. Plants adopting this strategy produce (at least) two morphs; a “high risk” seed morph, with a high mean fitness but high risk of reproductive failure, as well as a “low risk” seed morph, with a lower mean fitness and a lower risk of failure (Venable and Lawlor, 1980; Ellner, 1986; Baskin et al., 2013). Some systems also involve intermediate morphs. The most well-known examples of the HRLR pattern are systems that produce a “colonizer” seed morph optimized for dispersal (being light, having low seed dormancy, etc.) and a “maintainer” morph optimized for local establishment (see: Venable and Lawlor, 1980; Sendek et al., 2015). For example, in the annual herb *Crepis sancta* (Asteraceae), Imbert et al. (1997) observed that achenes produced near the center of the fruiting head had a pappus and were relatively small, whereas those produced on the periphery lacked a pappus and were on average twice as heavy as central achenes. These seed morphs were found to have distinct ecological functions—light (central) seeds with a pappus were dispersed by the wind, while heavy (peripheral) seeds, with larger embryos and energy stores, were found to have a strong establishment advantage in both intra- and interspecific competitions. While the predominant form of seed heteromorphism is to have two seed morphs (e.g., the two-lobed seeds of *Cakile maritima* and *C. edentula*, [see Maun and Payne (1989) and Donohue (1997)] there are some cases where plants produce more than two—e.g., *Heterosperma pinnatum* (Venable et al., 1987, 1995), *Salsola ferganica* (Ma et al., 2018) or the Amaranthaceae species *Atriplex sagittata* (Mandak and Pysek, 2005) and *A. aucheri* (Wang et al., 2015b). The stability of the ratio of seed morphs is found in many plant species, including *Triplasis purpurea* (Cheplick, 1996), *Crepis sancta* (Imbert and Ronce, 2001), and *Amphicarpaea edgeworthii*, and *A. bracteata* (Zhang et al., 2015).

Where heteromorphism is a consequence of diversifying bet-hedging, the most fundamental question is: how common should each morph be? Venable (1985) modeled the intrinsic rate of increase of a semelparous annual and solves for the conditions that are necessary and sufficient for a heteromorphic strategy to outcompete a monomorphic strategy, given assumptions concerning the expected intrinsic rates of increase of heteromorphic and monomorphic strategies for specific “year types,” the probability distribution of these year types, and the presence of constraints. This model has found considerable empirical support (e.g., Yang et al., 2017) and, although iterative, is computationally simple. However, this model is meant to compare the relative arithmetic fitnesses of monomorphic and heteromorphic seed strategies, and not to compare the relative fitness of different heteromorphic strategies (i.e., different combinations of seed morph proportions) or to quantify the degree of risk of equivalent heteromorphic strategies. Newer mathematical models have had notable success predicting the expected proportion of seed morphs, however these models have focused on quantifying the effect of innate and environmental factors (e.g., seed size, abiotic environmental cues, seed predator behavior, density-dependence, etc.) on the relative fitness of seed morphs, and not on optimal morph frequencies in general (Geritz, 1995; Venable et al., 1995; Braza et al., 2010; Yao et al., 2010; Wang et al., 2015a). Thus, to date the theoretical biological literature has not yet identified criteria by which different heteromorphic strategies can be compared.

In this paper, I aim to provide a basis for comparing the fitnesses of different heteromorphic strategies based on variation in fitness. Calculating the riskiness of different seed morph ratios—i.e., comparing seed heteromorphic strategies based on fitness and fitness variance—offers several notable advantages for understanding the evolutionary ecology of seed heteromorphism. First, it permits speculation on the impact that rare and severe events may have on different heteromorphic strategies. This is significant when there is a small, but nonetheless significant, chance of severely reduced (i.e., zero) fitness, since this results in extinction. In such cases, the best strategies should seek to reduce the risk of reproductive failure by reducing fitness variance, or by maximizing risk-adjusted fitness. An analogy can be made here with investing: when comparing any number of portfolios that offer equal returns, the one with the lower risk is the preferred investment. Thus, an optimal investment strategy will achieve the highest return possible adjusted for the risk of the loss of the initial capital. While the maximization of risk-adjusted utility is a well-studied problem in the field of mathematical finance (Markowitz, 1952; Merton, 1972), less theoretical work has been done on risk aversion in biological systems. Second, quantifying the relative riskiness of multiple seed heteromorphic strategies allows us to compare strategies to determine which ones minimize risk most effectively. This is significant when multiple solutions are possible to the model of Venable (1985)—such as when more than two morphs are produced, and therefore more than one combination of seed morphs can realize the same geometric fitness (e.g., as in Gairola et al., 2017). Third, the quantification of risk may also provide a useful basis by which to compare the riskiness of vegetative and sexual reproduction in seed plants.

Here I present a simple model that predicts these proportions using only parameters that can be estimated from experimental data, including the variability in fitness and the expected arithmetic mean fitness of each seed morph. I consider two optimality conditions, which relate to two kinds of fitness variance minimization: first, I consider the case where selection has simply minimized total fitness variance in fitness. Second, I consider the case where selection maximizes mean fitness gained per-unit of fitness variance gained. Using a given covariance matrix combined with fitness data adapted from a three-achene system in *Heterosperma pinnatum* described by Venable et al. (1995), I then use the model specified here to identify the proportions of three morphs that have the minimum risk or the maximum fitness-risk ratio. These findings may be generalizable to other systems where a single organism produces multiple types of offspring at once to minimize fitness variance [e.g., vegetative reproduction in the Solanaceae (Ewing, 1978; Winkler and Fischer, 2002)].

## Model

First, we consider the problem of a plant investing resources in two or more seed morphs with the goal of minimizing fitness variance. We explicitly consider a semelparous annual plant as an illustrative case, but this model could easily be adapted to consider the perennial case by treating fitness as spread out over multiple years. Several initial assumptions are made. First, I define risk as minimizing variance in fitness for a given degree of expected fitness. Second, I assume that there is a consistent relationship between the investment of resources and offspring fitness. Third, I must assume independence between the production of seed morphs; that is, I assume that resources spent on one seed morph cannot also be used to produce or improve other seed morphs. I then define the following variables.

## Definitions

- *m*_1_, *m*_2_ … *m*_*n*_ are defined as seed morphs—i.e., those structures that may result in the production of natural offspring by seed.
- *E*(*x*_*i*_) is the expected fitness of an investment of *x* resources in seed morph *i*
- *p*_*i*_ is the proportion of resources invested in seed morph *i*. The sum of all seed morph proportions must be equal to 1.
- σ_*i*_ is the standard deviation of the fitness of seed morph *i*
- σ_*ij*_ is the covariance between the fitnesses of seed morphs *i* and *j*
- *r*_*ij*_ is the correlation between the variance in fitness of seed morphs *i* and *j*

We then derive the optimal proportion of seed morphs for species subject to a simple risk-minimization strategy.

## Results

### Simple fitness variance minimization

Consider first the situation where there is only a single seed morph, *m*_1_. In this case, there is only one possible proportion of seed morphs, *p*_1_ = 1. The variance of the fitness of this single morph${\;\sigma }_{1}^{2}$ is by definition equal to the variance of the organism's total fitness${\;\sigma }_{T}^{2}$. Next, there are variety of cases in which there are two seed morphs, and many possible proportions of morphs. The variance in total fitness can be found by first letting the standard deviations of the two seed morphs *m*_1_ and *m*_2_ be σ_1_ and σ_2_ so that the standard deviation of an organisms' total fitness is:

(1.1) $$\begin{array}{l} \sigma_{T}=p_{1}\sigma_{1}+p_{2}\sigma_{2} \end{array}$$

Squaring Eq. (1.1) yields the variance in total fitness.

(1.2) $$\begin{array}{l} \sigma_{T}^{2}=p_{1}^{2}\sigma_{1}^{2}+p_{2}^{2}\sigma_{2}^{2}+2p_{1}p_{2}\sigma_{12} \end{array}$$

And σ_12_ represents the covariance between *m*_1_ and *m*_2_ such that:

(1.3) $$\begin{array}{l} \sigma_{12}=r_{12}\sigma_{1}\sigma_{2} \end{array}$$

Here *r*_12_ is the correlation between the fitness variance of the seed morphs *m*_1_ and *m*_2_.

### Fitness variance minimization for a heteromorphic strategy with two morphs

Next, I determine the optimal weighting of the various seed morphs in order to minimize fitness variance. One possibility can be solved easily. Consider first the situation where the fitness variances of the two seeds are perfectly positively correlated (i.e., *r*_12_ = 1). If the two variances are exactly equal, or if there is no selection with respect to variance, there is no meaningful difference in allocating resources to *m*_1_ instead of *m*_2_ or vice versa from an optimization perspective, since the risk of each is the same. Consider next the situation where the fitness variance of the two seed morphs are perfectly negatively correlated (i.e., *r*_12_ = −1). In this case, a perfect diversification bet-hedging strategy is in principle possible, since high variability in the fitness of one morph will be offset by reduced variability in the other. So, the fitness variance in this case is:

(2.1) $$\begin{array}{l} \sigma_{T}^{2}={\left(p_{1}\sigma_{1}-p_{2}\sigma_{2}\right)}^{2} \end{array}$$

Fitness variance is optimally zero, so the proportions when the total variance, and hence the total standard deviation, is equal to zero, can be found. First:

$$\begin{array}{l} \sigma_{T}=p_{1}\sigma_{1}-p_{2}\sigma_{2}=0\\ p_{1}\sigma_{1}=p_{2}\sigma_{2} \end{array}$$

By definition, *p*_*T*_ = 1 = *p*_1_ + *p*_2_. Therefore *p*_2_ = 1−*p*_1_.

$$\begin{array}{l} p_{1}\sigma_{1}=\left({1-p}_{1}\right)\sigma_{2}\\ p_{1}\left(\sigma_{1}+\sigma_{2}\right)=\sigma_{2} \end{array}$$

Therefore:

(2.2) $$\begin{array}{l} p_{1}=\frac{\sigma_{2}}{\sigma_{1}+\sigma_{2}} \end{array}$$

And *p*_2_ = 1−*p*_1_. These are the optimal proportions for two seed morphs whose fitness variances are perfectly negatively correlated. However, this is an extreme case. It is more biologically realistic, given the positive but imperfect correlations between different physiological and developmental processes that affect seed production and maturation, as well as the environmental conditions that affect them, to expect that the correlation between the variances in fitness of different seed morphs will fall somewhere between−1 and +1. Thus, for the optimal proportion of each morph in this case:

(2.3) $$\begin{array}{l} \sigma_{T}^{2}={\left(p_{1}\sigma_{1}+p_{2}\sigma_{2}\right)}^{2} \end{array}$$

Since *p*_2_ = 1−*p*_1_:

$$\begin{array}{l} \sigma_{T}=\sqrt{p_{1}^{2}\sigma_{1}^{2}+{\left(1-p_{1}\right)}^{2}\sigma_{2}^{2}+2p_{1}\left(1-p_{1}\right)\sigma_{12}}\\ \sigma_{T}=\sqrt{p_{1}^{2}\sigma_{1}^{2}+\sigma_{2}^{2}+p_{1}^{2}\sigma_{2}^{2}-2p_{1}\sigma_{2}^{2}+2p_{1}\sigma_{12}-2p_{1}^{2}\sigma_{12}} \end{array}$$

Next, by taking the partial derivative of this expression with respect to *p*_1_, we obtain:

$$\begin{array}{l} \frac{\partial \sigma_{T}}{\partial p_{1}}=\frac{2p_{1}\sigma_{1}^{2}+2p_{1}\sigma_{2}^{2}-2\sigma_{2}^{2}+2\sigma_{12}-4p_{1}\sigma_{12}}{2\cdot \sqrt{p_{1}^{2}\sigma_{1}^{2}+\sigma_{2}^{2}+p_{1}^{2}\sigma_{2}^{2}-2p_{1}\sigma_{2}^{2}+2p_{1}\sigma_{12}-2p_{1}^{2}\sigma_{12}}} \end{array}$$

This can be simplified, and the resulting expression can be set equal to zero to find the proportions of each morph that correspond to the case when fitness variance is minimized.

$$\begin{array}{l} \frac{p_{1}\sigma_{1}^{2}+p_{1}\sigma_{2}^{2}-\sigma_{2}^{2}+\sigma_{12}-2p_{1}\sigma_{12}}{\sqrt{p_{1}^{2}\sigma_{1}^{2}+\sigma_{2}^{2}+p_{1}^{2}\sigma_{2}^{2}-2p_{1}\sigma_{2}^{2}+2p_{1}\sigma_{12}-2p_{1}^{2}\sigma_{12}}}=0 \end{array}$$

This expression is simplified by multiplying the right side by the denominator.

$$\begin{array}{l} p_{1}\sigma_{1}^{2}+p_{1}\sigma_{2}^{2}-\sigma_{2}^{2}+\sigma_{12}-2p_{1}\sigma_{12}=0\\ p_{1}\left(\sigma_{1}^{2}+\sigma_{2}^{2}-2\sigma_{12}\right)=\sigma_{2}^{2}-\sigma_{12} \end{array}$$

Therefore:

(2.4) $$\begin{array}{l} p_{1}=\frac{\sigma_{2}^{2}-\sigma_{12}}{\sigma_{1}^{2}+\sigma_{2}^{2}-2\sigma_{12}} \end{array}$$

And *p*_2_ = 1−*p*_1_. These are the proportions of the two seed morphs that minimize variance in fitness, when the correlation between the fitness variance of the two seed morphs is smaller than 1 but greater than−1. Thus, optimal proportions can be computed solely by finding the standard deviations of the two seed morphs and the covariance between them.

### Maximization of the fitness-risk ratio for a heteromorphic strategy with two morphs

However, minimizing the total variance in fitness without considering the fitnesses of the two seed morphs assumes that the fitnesses of the seed morphs are approximately equal, or at the very least, not highly unequal. However, this assumption ignores the possibility that an optimal risk reduction strategy may include both a high variance morph and a low variance morph. In nature, this it is common to find a “non-disperser” seed morph with low fitness variance and moderate fitness in all environments, as well as a “disperser” seed morph, with high fitness variance and either very low or very high fitness, depending on its establishment success in a new, uncolonized environment (De Clavijo, 2001; Lerner et al., 2008; Dubois and Cheptou, 2012). For these cases, as well as others where morph fitnesses are unequal, the optimal strategy may not be merely to minimize overall variance in fitness but instead to maximize “risk-efficiency,” the amount of fitness accrued per unit of variance in fitness gained. Risk-efficiency can be quantified by expressing the relationship between the expected fitness of a given amount of available resources as the fitness-risk ratio *R*_*T*_, where *E*(*x*_*T*_) represents the expected fitness gained from investing all available resources (*x*_*T*_) in one or more seed morphs. The maximization of this expression can be represented mathematically as:

(3.1) $$\begin{array}{l} \underset{p}{\max}R_{T}=\frac{E\left(x_{T}\right)}{\sigma_{T}} \end{array}$$

The expected fitness function for the respective fitnesses of seed morphs *m*_1_ and *m*_2_ can then be calculated—let these values be represented by *w*_1_ and *w*_2_. Note that these fitnesses represent the geometric mean fitnesses of the two morphs (i.e., the *n*^th^ root of the product of *n* fitness measurements, made across generations), not the arithmetic mean fitnesses. We obtain:

(3.2) $$\begin{array}{l} E\left(x_{T}\right)=p_{1}w_{1}+p_{2}w_{2} \end{array}$$

That is, the expected total fitness is equal to the weighted sum of the fitnesses of the individual seed morphs. The standard deviation of the expected total fitness can be written as:

(3.3) $$\begin{array}{l} \sigma_{T}=\sqrt{p_{1}^{2}\sigma_{1}^{2}+p_{2}^{2}\sigma_{2}^{2}+2p_{1}p_{2}\sigma_{12}} \end{array}$$

*R*_*T*_ can be rewritten as:

(3.4) $$\begin{array}{l} R_{T}=\frac{p_{1}w_{1}+p_{2}w_{2}}{\sqrt{p_{1}^{2}\sigma_{1}^{2}+p_{2}^{2}\sigma_{2}^{2}+2p_{1}p_{2}\sigma_{12}}} \end{array}$$

Because the sum of the two proportions is by definition equal to 1, identifying either is sufficient to solve for both. To find the proportion *p*_1_ that maximizes *R*_*T*_, we take the partial derivative with respect to *p*_1_, then set the resulting expression equal to zero to find the value where the fitness-risk ratio is minimized (i.e., the critical value of the function).

(3.5) $$\begin{array}{l} \frac{\partial S_{T}}{\partial p_{1}}=\;p_{1}w_{1}+p_{2}w_{2}\;\cdot \;\left(-\frac{1}{2}\right)\;\cdot \;{\left(p_{1}^{2}\sigma_{1}^{2}+p_{2}^{2}\sigma_{2}^{2}+2p_{1}p_{2}\sigma_{12}\right)}^{-\frac{3}{2}}\cdot 2p_{1}\sigma_{1}^{2}\\ \;+2p_{2}\sigma_{12}+{\left(p_{1}^{2}\sigma_{1}^{2}+p_{2}^{2}\sigma_{2}^{2}+2p_{1}p_{2}\sigma_{12}\right)}^{-\frac{1}{2}}\;\cdot \;w_{1} \end{array}$$

To simplify this expression, we multiply throughout by $(p_{1}^{2}\sigma_{1}^{2}+p_{2}^{2}\sigma_{2}^{2}+2p_{1}p_{2}\sigma_{12})\frac{1}{2}$. This yields:

$$\begin{array}{l} \frac{\partial S_{T}}{\partial p_{1}}=-\frac{\left(p_{1}w_{1}+p_{2}w_{2}\right)\left(p_{1}\sigma_{1}^{2}+p_{2}\sigma_{12}\right)}{p_{1}^{2}\sigma_{1}^{2}+p_{2}^{2}\sigma_{2}^{2}+2p_{1}p_{2}\sigma_{12}}\;+w_{1} \end{array}$$

The *w*_1_ term can be brought to the numerator and the resulting expression can be set equal to zero to find the optimum proportion of *m*_1_.

$$\begin{array}{l} -\frac{\left(p_{1}w_{1}+p_{2}w_{2}\right)\left(p_{1}\sigma_{1}^{2}+p_{2}\sigma_{12}\right)+w_{1}\left(p_{1}^{2}\sigma_{1}^{2}+p_{2}^{2}\sigma_{2}^{2}+2p_{1}p_{2}\sigma_{12}\right)}{p_{1}^{2}\sigma_{1}^{2}+p_{2}^{2}\sigma_{2}^{2}+2p_{1}p_{2}\sigma_{12}}=0 \end{array}$$

The denominator is removed by bringing it to the right side, and the expression is rearranged.

$$\begin{array}{l} w_{1}\left(p_{1}^{2}\sigma_{1}^{2}+p_{2}^{2}\sigma_{2}^{2}+2p_{1}p_{2}\sigma_{12}\right)-\left(p_{1}w_{1}+p_{2}w_{2}\right)\left(p_{1}\sigma_{1}^{2}+p_{2}\sigma_{12}\right)=0\\ {w_{1}p}_{2}^{2}\sigma_{2}^{2}+w_{1}p_{1}p_{2}\sigma_{12}-w_{2}p_{1}p_{2}\sigma_{1}^{2}-w_{2}p_{2}^{2}\sigma_{12}=0 \end{array}$$

Substitute (1−*p*_1_) for *p*_2_ and rewrite as:

$$\begin{array}{l} w_{1}\sigma_{2}^{2}-w_{2}\sigma_{12}=p_{1}(w_{2}\sigma_{1}^{2}-w_{1}\sigma_{12}+w_{1}\sigma_{2}^{2}-w_{2}\sigma_{12}) \end{array}$$

And *p*_1_ can be solved for directly:

(3.6) $$\begin{array}{l} p_{1}=\frac{{\;w}_{1}\sigma_{2}^{2}-w_{2}\sigma_{12}}{w_{1}\sigma_{2}^{2}+w_{2}\sigma_{1}^{2}-\left(w_{1}+w_{2}\right)\sigma_{12}} \end{array}$$

And *p*_2_ = 1−*p*_1_. These are the optimal proportions of the seed morphs that maximize the ratio of fitness to fitness variance in an organism with two seed morphs of unequal fitness. Note that if *w*_1_ = *w*_2_, this expression simplifies to Eq. (2.4). To calculate optimal proportions of seed morphs, the only quantities that must be estimated or measured are the mean (geometric) fitnesses of each morph, the fitness variances of each morph, and the fitness covariance of the two morphs.

### Maximization of the fitness-risk ratio for a heteromorphic strategy with n morphs

The method used in Section 3 can be generalized to solve for the case where there are more than two seed morphs. Here the fitness-risk ratio is maximized for *n* morphs. The assumptions made here are the same as for previous calculations, and the total fitness of a parent plant is made up of seed morphs *m*_1_, *m*_2_ … *m*_*n*_. In this case the expected total fitness is:

(4.1) $$\begin{array}{l} E\left(x_{T}\right)=w_{1}r_{1}+w_{2}r_{2}+\ldots w_{n}r_{n} \end{array}$$

First, the optimal proportions *p*_1_, *p*_2_ … *p*_*n*_ are defined as values in an optimal proportion vector *p* and the individual fitnesses *w*_1_, *w*_2_ … *w*_*n*_ as values in a fitness return vector *w*.

$$\begin{array}{l} p=\;\left[\begin{array}{c} \begin{array}{c} p_{1}\\ p_{2}\\ \cdots \end{array}\\ p_{n} \end{array}\right]\;and\;w=\left[\begin{array}{c} \begin{array}{c} w_{1}\\ w_{2}\\ \cdots \end{array}\\ w_{n} \end{array}\right] \end{array}$$

The expected total fitness of an investment of *x_T_* resources is then defined as:

(4.2) $$\begin{array}{l} E\left(x_{T}\right)=p^{T}w \end{array}$$

The standard deviation of total fitness is:

$$\begin{array}{l} \sigma_{T}=\sqrt{p^{T}Vp} \end{array}$$

Thus, for *p*^*T*^ = 1, where *V* is the variance-covariance matrix, the fitness risk ratio is written as:

(4.3) $$\begin{array}{l} \underset{p}{\max}S_{T}=\frac{p^{T}w}{\sqrt{p^{T}Vp}} \end{array}$$

Since *p*^*T*^ · *p* = *p*^2^, the derivative of the fitness-risk ratio with respect to seed morph proportion yields:

$$\begin{array}{l} \frac{\partial S_{T}}{\partial p}=p^{T}w\left(-\frac{1}{2}\right){\cdot \left(p^{T}Vp\right)}^{-\frac{3}{2}}\cdot 2pV+\frac{w}{\sqrt{p^{T}Vp}} \end{array}$$

The−3/2 exponent is eliminated by multiplying through by $\sqrt{p^{T}Vp}.$

$$\begin{array}{l} \frac{\partial S_{T}}{\partial p}=p^{T}w\cdot [-\left(p^{T}Vp\right)-1\cdot pV]+w \end{array}$$

And the expression is then written in terms of r and is set equal to zero.

$$\begin{array}{l} p^{T}w\cdot [-{\left(p^{T}Vp\right)}^{-1}\cdot pV]+w=0\\ \;w=\frac{p^{T}w\cdot pV}{p^{T}Vp} \end{array}$$

The expression $\frac{p^{T}w}{p^{T}Vp}$ represents the price of risk “R”. This quantity describes how much fitness is gained for a given increase in the variance of fitness. The price of risk is the fitness divided by the variance in fitness; it tells us how much fitness is gained per unit of volatility in fitness. Thus, we obtain:

$$\begin{array}{l} w=RpV \end{array}$$

The variance-covariance matrix *V* is eliminated by multiplying both sides of the equation by its inverse *V*^−1^.

$$\begin{array}{l} V^{-1}w=Rp \end{array}$$

Each side of this equation can be defined as a vector, *z*. Thus:

(4.4) $$\begin{array}{l} {z=V}^{-1}w=Rp \end{array}$$

Eq. (4.4) is interesting since *z* varies in proportion with *p*. However, the solution of *z* can be found by measuring fitnesses to calculate the components of the vector *w*, as by measuring fitness variances to calculate the components of the inverse of the variance-covariance matrix *V*. These *z* values can then be used to solve for *p*. Thus, the equation for each *p*_*i*_ in the vector *p* can be written as the normalized *z*:

(4.5) $$\begin{array}{l} p_{i}=\frac{z_{i}}{\sum_{i=1}^{n}z_{i}} \end{array}$$

This is the equation for the optimal proportion of seed morphs in a system with *p* seed morphs. For example, in a three-morph system:

(4.6) $$\begin{array}{l} p_{1}=\frac{z_{1}}{z_{1}+z_{2}+z_{3}} \end{array}$$

### Identifying the proportions of seed morphs that minimize total risk or maximize the fitness risk-return ratio

These equations obtained above can be used to directly compare different seed heteromorphic strategies. For example, Venable et al. (1995) studied the evolutionary ecology of seed morphs in Mexican populations of the selfing annual *Heterosperma pinnatum*. This species has no seed bank and produces three achene types—central, peripheral, and intermediate—which differ in morphology and dormancy, but not in size or growth rate. Peripheral achenes are short, wide, and winged, while central achenes are long, narrow, and awned and intermediate achenes have a mixture of both traits. Moreover, central achenes generally have lower seed dormancy than peripheral or intermediate achenes. Together these traits confer high dispersivity to central achenes, but also make them vulnerable to adverse conditions such as heavy rains (Venable et al., 1987). In the Tula, Hidalgo population described in Venable et al. (1995), the relative (standardized) fitnesses of the central, intermediate, and peripheral achenes were 0.69, 0.98, and 1.0, respectively. The authors attributed the low fitness of the central achenes, at least in part, to high early-season precipitation at the Tula site; the implication is that the central achene is a high-risk, high-reward morph, while the peripheral morph is a low-risk, low-reward morph, and the intermediate morph is somewhere in between.

The geometric fitnesses of the different morphs and the corresponding covariance matrix are not provided in Venable et al. (1995). However, consider a theoretical experiment that yielded the following arbitrarily scaled expected fitnesses for high risk-high reward (HRHR), intermediate risk-intermediate reward (IRIR), and low risk-low reward (LRLR) seed morphs: *w*_*HRHR*_ = 4, *w*_*IRIR*_ = 2, and *w*_*LRLR*_ = 1 as well as the following covariance matrix *V*.

$$\begin{array}{l} V=\left[\begin{array}{c} 0.02\;0.0005\;0.0005\\ 0.0005\;0.010.0005\\ 0.00050.00050.005 \end{array}\right]. \end{array}$$

**Figure 1:**
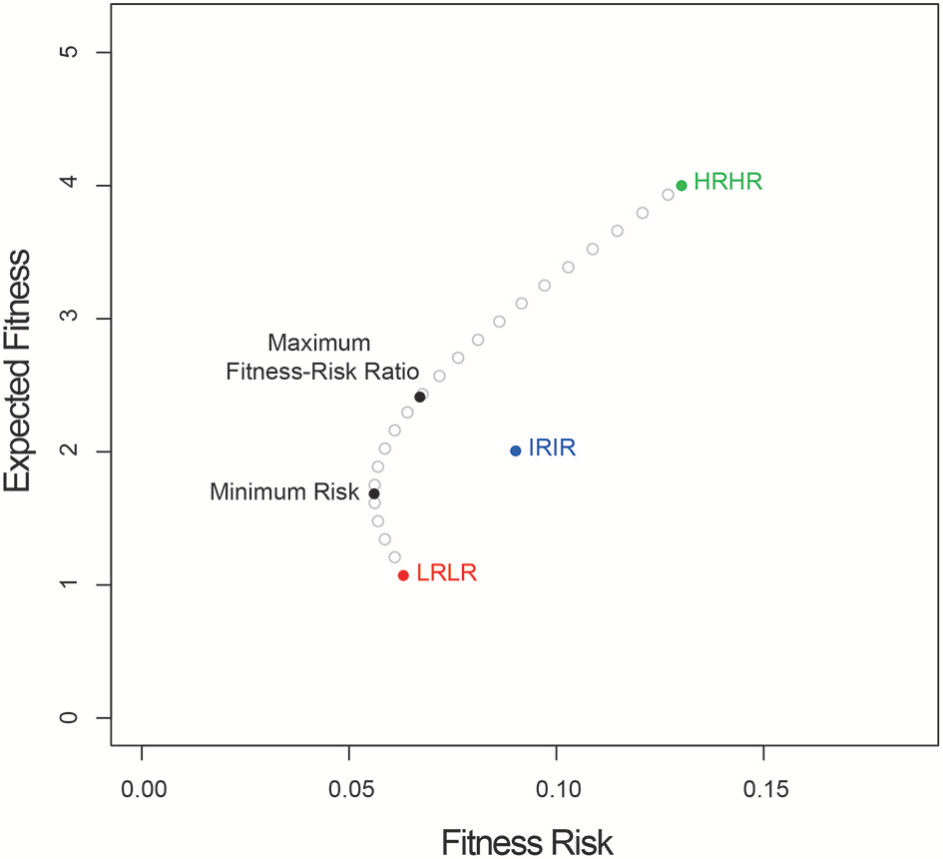
Expected fitness vs. fitness risk for a three-morph heteromorphic system. Shown are the expected fitness and risk for three “pure” strategies: HRHR (high-risk, high-reward), IRIR (intermediate risk, intermediate reward), and LRLR (low risk, low reward), as well as for the strategies that minimize total risk or maximize the expected fitness-risk ratio.

Here, the fitness and variance of the high risk-high return morph are highest, and the corresponding values for the low risk-low return morph are lowest, with the intermediate morph in between. In this example, the covariances between the expected fitnesses of the morphs are equal. Figure 1 shows a plot of the curve specifying the minimum risk at each expected fitness, as well as points indicating: (1) the expected fitnesses of each of the three monomorphic strategies; (2) the expected fitness of the solution to Equation (2.4), which indicates the heteromorphic strategy with minimum total risk; and (2) the expected fitness of the solution to Equation (4.6), which indicates the heteromorphic strategy that offers the greatest expected fitness per unit of additional fitness risk. In this case, the proportions of seed morphs that minimize total risk are: HRHR = 0.135; IRIR = 0.278; LRLR = 0.587, and the proportions that maximize the fitness-risk ratio are: HRHR = 0.358; IRIR = 0.340; LRLR = 0.302. While the numbers used here are arbitrary, a graph similar to Figure 1 can be plotted for any series of seed morphs.

## Discussion

Here I provide equations that permit the direct comparison between different seed heteromorphic strategies based on variation in fitness. Simply put, just as a heteromorphic strategy with a higher geometric mean fitness should be favored over one with lower fitness, among strategies with similar or identical fitnesses the one that more effectively minimizes fitness variance is better insulated from severe reductions in fitness that may occur randomly and thereby may have an advantage over long periods of time. The equations introduced here permit the computation of the risk-return ratio, which is a simple estimate the risk-adjusted fitness performance of a given strategy. These equations may be useful for evolutionary ecologists interested in seed heteromorphism because they permit the direct comparison of multiple heteromorphic strategies, and therefore provide the ability to make predictions about any plant species showing seed heteromorphism. The main prediction is that even when two (or more) strategies are fitness-equivalent, heteromorphic strategies with higher risk-return ratios should outcompete those with lower risk-return ratios because the riskier strategies have increased exposure to rare, fitness-reducing events that may causing severe fitness loss. Where seed morphs at a given expected fitness exceed the minimum level of risk, there may exist evolutionarily relevant physiological or ecological constraints to doing so.

The derivation of these equations advances our theoretical understanding of seed heteromorphism in three main ways. First, and most generally, these equations present a criterion by which we can identify which heteromorphic strategies are more or less risky. Here risk refers to the variance in offspring fitness, meaning that high-risk heteromorphic strategies are more vulnerable than fitness-equivalent low-risk strategies to rare events that may dramatically lead to extinction. This agrees with previous theoretical work—Gillespie (1974, 1977) proposed that natural selection should, all things being equal, favor strategies that minimize variance in offspring fitness (i.e., risk; see also: Starrfelt and Kokko, 2012). This principle has been corroborated by models showing a small but decisive advantage for risk-averse strategies even when they had equivalent fitnesses (Kolodny and Stern, 2017). This difference is especially important when considering how seed morph proportions should evolve in natural systems that feature very rare events that impose substantial fitness costs on high-risk seed morphs (e.g., severe droughts). According to Gillespie's risk-minimization principle, heteromorphic strategies that show lower fitness risk-return ratios should be less likely to suffer severe fitness losses from such events. The equations presented here provide the first method to empirically test these predictions in plant species showing seed heteromorphism.

Second, by quantifying the risk profiles of heteromorphic strategies, these equations are the first published basis by which seed heteromorphic strategies that contain more than two morphs can be compared. Given the prevalence of multi-morph seed strategies in nature and the fact that they remain—relative to two-morph strategies—relatively understudied, this is a straightforward advance that should be of considerable benefit to future studies of strategies that include many morph types.

Third, it may be the case that other forms of risk-spreading reproductive strategies—such as the production of both sexual offspring and vegetatively propagated stolons—are also well-described by equations that measure the inherent riskiness of different reproductive modes, especially when variable risk has been shown to be an intrinsic feature of a plant's ecological niche (Thompson and Beattie, 1983; Winkler and Fischer, 2002). In such a situation, the risk profile of a reproductive strategy of a plant that produced two seed morphs and one stolon could be directly compared to the risk profile of a plant that produced three different seed morphs. Such comparisons, which require both further theoretical consideration and empirical validation, may help shed light on the ecological dynamics surrounding the trade-offs between vegetative and sexual reproduction in plants.

Although this model is meant to act as an efficient null hypothesis for optimal resource allocation, the predictive validity of this model will likely be limited by system-specific factors such as architectural effects (Ortiz et al., 2009) and allometry (Lei et al., 2010). In addition, this model assumes density independence; although bet-hedging strategies are supposed to involve in highly stochastic environments, where cues may be of limited importance, there is considerable empirical work suggesting that density dependence affects the relative fitness of seed morphs (Donohue, 1997; Koyama, 1998). Other important caveats are that it may be difficult to directly measure the proportion of resources invested in different seed morphs, and that investment in seed output may be affected a general growth-maintenance-reproduction developmental program. Moreover, as in many models, pleiotropy and other genetic constraints may affect the ability of a plant to independently vary investment in different seed morphs, since their production is likely to involve many common developmental pathways. Lastly, in nature pure evolutionary strategies are rare, and many seed-heteromorphic species show evidence of both bet-hedging and phenotypic plasticity (Lu et al., 2012; Auld and Rubio, 2013). Providing direct empirical support for a bet-hedging hypothesis of ecological traits is often difficult, usually because the predictions needed to provide a clear test can be difficult to determine. Nevertheless, while these limitations may affect the specificity of predictions, it is hoped that this generalizable null model is useful in the majority of species that have evolved seed heteromorphism.

## Data availability

All relevant data is contained within the manuscript.

## Author contributions

The author confirms being the sole contributor of this work and has approved it for publication.

### Conflict of interest statement

The author declares that the research was conducted in the absence of any commercial or financial relationships that could be construed as a potential conflict of interest.
